# Development of Apomictic 56-Chromosomal Maize–*Tripsacum* Hybrids: A Potential Breakthrough in Heterosis Fixation

**DOI:** 10.3390/plants13152138

**Published:** 2024-08-01

**Authors:** Viktor Andreevich Sokolov, Pavel Alexandrovich Panikhin, Kirill Olegovich Plotnikov, Grigory Yurievich Chepurnov, Alexander Genadievich Blinov

**Affiliations:** 1 Laboratory of Plant Cytology and Apomixis, Institute of Molecular and Cell Biology, Siberian Branch of the Russian Academy of Sciences, Academician Lavrentyev Avenue, 8/2, 630090 Novosibirsk, Russia; 2 Laboratory of Food Plants Introduction, Central Siberian Botanical Garden, Siberian Branch of the Russian Academy of Sciences, Zolotodolinskaya Street, 101, 630090 Novosibirsk, Russia; 3 Sector of Molecular Genetic Principles of Regeneration, Institute of Cytology and Genetics, Siberian Branch of the Russian Academy of Sciences, Akademika Lavrentieva Avenue, 10, 630090 Novosibirsk, Russia; 4 Laboratory of Agricultural Plant Biotechnology, Siberian Research Institute of Plant Growing and Breeding, C-200 Avenue, 5/1, 630501 Krasnoobsk, Russia

**Keywords:** *Tripsacum dactyloides*, *Zea mays*, hybrid, apomixis, *Pox3*, *trnL*

## Abstract

Maize (*Zea mays* L.) is one of the most demanded grain crops in the world. Currently, production has exceeded one billion tons and is increasing by 3–5% annually. Such growth is due to the genetic potential of the crop and the use of heterosis F1 hybrids in production. However, the need to produce first-generation seed annually poses significant challenges and is an economically costly technology. A solution to this problem may be the transfer of the asexual (apomictic) mode of reproduction to maize from its wild relative, eastern gamagrass (*Tripsacum dactyloides* L.). In this work, we report the production of 56-chromosome apomictic hybrids of maize (*Zea mays* L.) with eastern gamagrass (*T. dactyloides* L.) with restored anther fertility. The mode of reproduction of the plant was confirmed by counting chromosomes and sequencing the nuclear gene (*Pox3*) and chloroplast tRNA-Leu (*trnL*) gene. These apomictic hybrids had karyotypes of 2n = 56 = [(10Zm(573MB) + 36Td) + 10Zm(611CB)] and 2n = 56 = [(10Zm(611CB) + 36Td) + 10Zm(611CB)]. The resulting hybrids can be widely used as a fodder crop.

## 1. Introduction

In recent decades, maize (*Zea mays* L.) has become the most in-demand cereal crop in the world [1]. Since 2010, its world production has exceeded one billion tons and has been increasing annually by 3–5% [2]. This is primarily due to the genetic potential of the crop and the use of heterosis F1 hybrids in production. At the same time, the need for the annual production of first-generation seeds creates significant problems and is an economically costly technology, both in terms of the use of sowing areas and its scientific support. Therefore, even at the early stages of hybrid breeding introduction in the 1930s, M.S. Navashin and G.D. Karpechenko [3] suggested the idea of the possible fixation of heterosis by cloning hybrid plants in a number of generations. The transfer of the asexual mode of reproduction through seeds [4] (apomictic) to maize was supposed to be from a wild relative—eastern gamagrass (*Tripsacum dactyloides* L.)—which is a perennial plant. However, it was possible to begin to find an experimental solution to this problem only after the organization of the Siberian Branch of the USSR AS in Novosibirsk in 1957. The first apomictic maize–*Tripsacum* hybrid was obtained in 1964 [5]. Unfortunately, the genetic control of the trait of seedless reproduction turned out to be much more complicated [6] than it was believed at the beginning of the research [7]. The study of the hybrids of different genomic compositions has shown that the presence of at least nine specific gamagrass chromosomes is necessary for them to exhibit apomixis [8]. With this amount of genetic material from the wild parent, it is impossible to obtain plants with the maize habitus. In addition, apomixis in *T. dactyloides* is pseudogamous, and central cell fertilization is required for the formation of viable seeds. At the same time, the hybrids created were male-sterile and required hand pollination with maize to set seed. Various attempts to obtain plants capable of producing pollen based on the materials created by D.F. Petrov and his colleagues were unsuccessful [9]. This problem was faced by all researchers who tried to use the unique genetic potential of gamagrass to expand the breeding diversity of maize. They agreed that hybrids between these forms exhibit female fertility but do not form pollen [10,11]. Our studies conducted with the material obtained by D.F. Petrov’s group gave the same results regardless of the ratio of the genomes of the parental forms [12,13,14].

Thanks to the spread of molecular genetics techniques, researchers have gained more information about the mechanisms of the genetic control of apomixis. The genes *APOLLO* (*apomixis-linked locus*—from *Boechera*), *HpARI* (*ARIADNE7*—from Hypericum), *PsORC3a* (*ORIGIN RECOGNITION COMPLEX*—from Paspalum), and *ASGR-BBML* (*Apospory-Specific Genomic Region-BabyBooM-Like*—from Pennisetum) show a close genetic relationship with the apomixis locus in many plant species and are the main candidate genes controlling this trait [15,16,17,18]. However, despite significant progress, researchers have no unified model for controlling apomixis, and the transmission of this trait to cultivated plants from their wild relatives or ancestors is extremely difficult at this time [19]. For *T. dactyloides*, there were no reports of apomixis being linked to the listed genes, but one dominant locus was found [20,21], which was mapped using RFLP markers. It was noted that although apomixis in *T. dactyloides* is inherited as a single Mendelian allele, it may in fact be controlled by a cluster of linked loci [20]. Therefore, the introgression of the apomixis locus from *Tripsacum* to maize is very difficult, which is also associated with the transmission of the trait to the next generation with low seed fertility and the poor development of viable adult plants [22].

Summarizing all the available data, we came to the conclusion that apomixis is a complex trait and it is practically impossible to transfer it by transferring genetic material with the possible conjugation of *Tripsacum* and maize chromosomes. However, the idea of obtaining fixed heterosis by transferring asexual reproduction to a cultivated plant is so attractive and economically significant that we decided to continue the research at a new qualitative level. Previously, it was already possible to obtain apomictic maize–*Tripsacum* hybrids, where one of the parents was tetraploid *Tripsacum* (72 chromosomes) [14]. However, male sterility remained a problem with these hybrids. With this in mind, we also used maize and tetraploid *Tripsacum* (which also produces significantly more pollen than diploid), but our objective was to select the genotypes of the hybrids capable of producing fertile pollen. Cytogenetically, such hybrids should be amphidiploid, as only in this case the normal conjugation of homologous chromosomes and the formation of viable pollen will be possible. These hybrids should have 56 chromosomes where haploid sets of chromosomes from maize lines used to produce commercial heterosis hybrids are combined. It was clear that a significant content of genetic material from gamagrass would affect cob and seed size. However, such plants would also have the potential advantages of the high bushiness, high cob number, and high forage protein quality of the wild relatives. In addition, with the high dynamism of parental genomes observed in hybrids, one can hope for the rapid breeding improvement of the resulting plants. Thus, the aim of this work was to obtain apomictic, 56-chromosomal maize–*Tripsacum* hybrids capable of producing fertile pollen.

## 2. Results

### 2.1. Obtaining 46-Chromosome Maize–Tripsacum Hybrids

**Figure 1:**
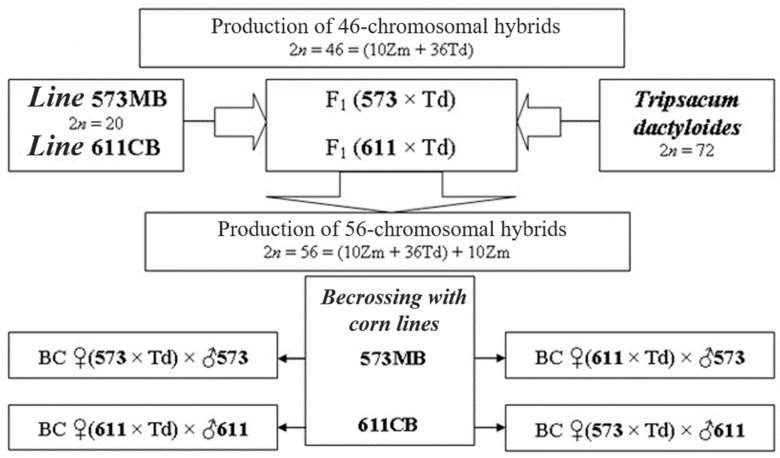
Scheme of stages of work for obtaining maize–*Tripsacum* hybrids.

At the first stage of the work, the maize hybrids with *Tripsacum* of genomic composition 2n = 46 = (10Zm + 36Td) were obtained (Figure 1). For this purpose, 169 cobs from line 573MB were pollinated with gamagrass pollen to obtain F1 hybrids (573MB × Td) and 124 cobs of line 611CB to obtain F1 hybrids (611CB × Td).

The analysis of hybridization results suggests that maize lines differ in the number of embryos and number of viable grains formed from their F1 plants (Table 1).

The differences in the number of embryos developed in the maize lines as a result of pollination were insignificant. However, taking into account the much smaller number of pollinated flowers in line 611CB, it is possible to understand its more successful hybridization with gamagrass relative to line 573MB. Nevertheless, the number of grains that formed the endosperm in line 611CB was an order of magnitude less compared to line 573MB, which indicates a higher abortivity of its embryos. As a result, the number of F1 plants obtained from line 573MB was 429, and from a similar cross with line 611CB was 3.

**Figure 2:**
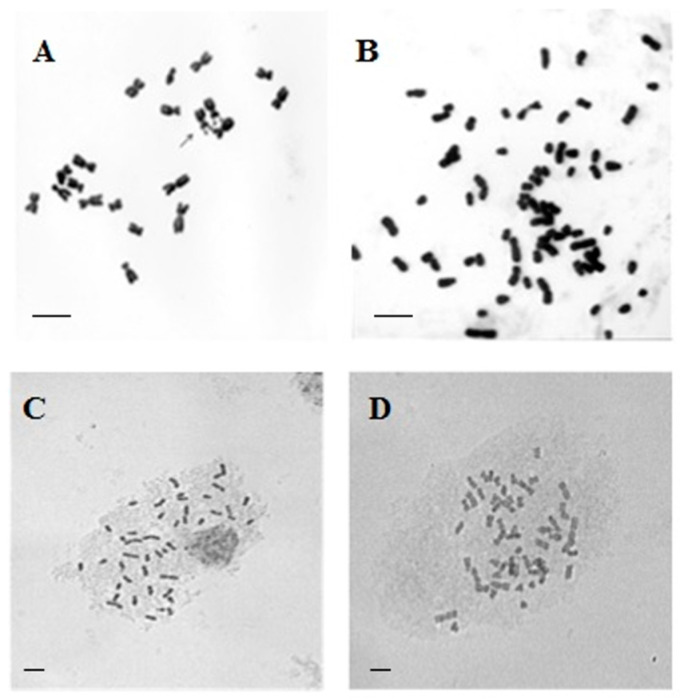
Metaphase plates: (**A**) *Zea mays* 2n = 20; (**B**) *Tripsacum dactyloides* 2n = 4x = 72; (**C**) maize–*Tripsacum* hybrid F1 ♀*Z. mays* × ♂*T. dactyloides* 2n = 46; (**D**) maize–*Tripsacum* hybrid BC1 ♀(*Z. mays* × *T. dactyloides*) × ♂*Z. mays* 2n = 56. In all the cases, the scale bar = 10 µm.

The analysis of karyotypes in the obtained plants showed the presence of two genomes from *Tripsacum* and one from maize—2n = 46. The number of parental genomes was determined by the number of kernel-forming chromosomes—two for *Tripsacum* and one for maize (see Section 4) (Figure 2). The resulting 46-chromosome forms were then backcrossed with parental maize lines to produce the seed progeny of BC1 (♀F1 × ♂573MB) and (♀F1 × ♂611CB) (Figure 1). Viable seeds were obtained from 20 F1 plants (573MB × Td) and 2 F1 plants (611CB × Td).

The analysis of the BC1 (♀F1 × ♂573MB) and BC1(♀F1 × ♂611CB) data from the pollination of 20 F1 plants (573MB × Td) (Table 2) showed that the F1 hybrid plants could be divided into two groups based on grain set: (1) Eleven plants exhibited a grain set of 1.02% and 0.75% when pollinated with the 573MB and 611CB lines, respectively. In absolute numbers, 23 grains per 2255 embryos were obtained for the BC1 combination (♀F1 × ♂573MB) and 40 grains per 5359 embryos for the BC1 combination (♀F1 × ♂611CB). (2) nine plants showed a grain set of 18.88% when pollinated with the 573MB line and 19.43% when pollinated with the 611CB line. In absolute numbers, 277 grains per 1467 embryos were obtained for the BC1 combination (♀F1 × ♂573MB) and 663 grains per 3412 embryos for the BC1 combination (♀F1 × ♂611CB). In BC1 from F1 (611CB × Td), 18 grains (3%) were obtained for two of the three plants when pollinated by the 611CB line and 6 grains (1.47%) when pollinated by the 573MB line.

### 2.2. Production of 56-Chromosome Maize–Tripsacum Hybrids

Progeny with the following karyotypes can be observed in BC1:

1. Plants produced by fertilization by a haploid maize sperm with an egg originating from a megaspore that has undergone meiosis and carries 18 *Tripsacum* chromosomes with maize chromosomes added is a BII hybridization;
2. The 56-chromosome plants, which result from BIII hybridization when an unreduced egg carrying 46 chromosomes is fertilized by a haploid maize sperm;
3. The 46-chromosome forms resulting from asexual reproduction.

**Figure 3:**
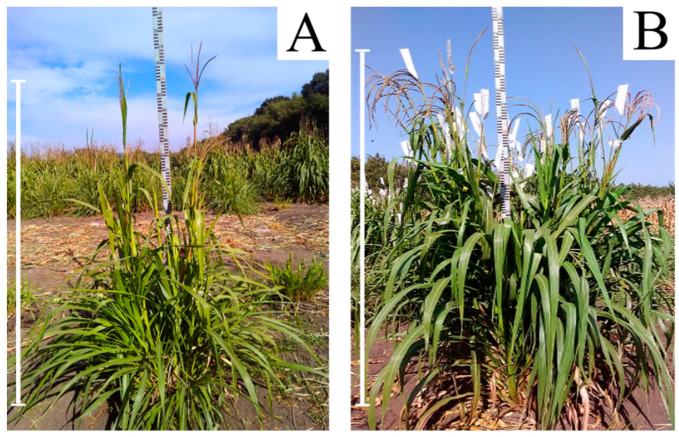
Plant habitus of (**A**) 46- and (**B**) 56-chromosome maize–*Tripsacum* hybrids. In all the cases, the scale bar = 2 m.

BII hybrids obtained by the pollination of F1 with pollen with maize lines are easily identified by the habitus of plants (Figure 3) and since they were not of interest for solving the task, we excluded them from our consideration.

The plants with karyotype 2n = 46 (4/Б10-9, Table 3) are the product of the asexual reproduction of the parental form. No BIII hybridization is observed in these crosses, but it is possible that with more backcross progeny they would have been detected. The study of the progeny of this plant is ongoing. The hybrid (3/Б10-9, Table 3), which gave only BIII progeny in the backcross progeny, is the product of the fertilization of an egg not capable of parthenogenetic development. This phenomenon is the result of the splitting of the traits of non-reduction and parthenogenesis in *Tripsacum*. Previously, this fact was observed in another series of crosses of the same form of gamagrass [23].

Since BIII hybridization occurs in apomictic plants at a frequency of approximately 1–3% [8], the finding of a ratio between the number of progeny with 56- and 46-chromosome plants among BC1s indicates the degree of apomixis expression in the F1 hybrids. Table 3 shows that along with apomictic, the cases of BIII hybridization were observed in all the five remaining F1s. However, two F1 plants, № 1/Б10-9 and 3/Б10-8, yielded BIII hybrids with the lowest frequency. Further backcrossing of the obtained 56-chromosomal hybrids BC1 with the pollen of maize lines showed that some hybrids had female sterility close to 100% and seed material from № 1/Б10-9 and № 3/Б10-1 was not obtained (Table 3). Stability in habitus and chromosome number in BC2 was exhibited by five 56-chromosome plants from № 3/Б10-8—one with karyotype 2n = 56 = [(10Zm(573MB) + 36Td) + 10Zm(573MB)] and four with karyotype 2n = 56 = [(10Zm(573MB) + 36Td) + 10Zm(611CB)] and one 56-chromosome plant from № 26/Б11-1 with karyotype 2n = 56 = [(10Zm(611CB) + 36Td) + 10Zm(611CB)]. The subsequent generations of these hybrids were tested for the stability of the expression of the asexual mode of reproduction in them.

### 2.3. Selection of Apomictic 56-Chromosomal Maize–Tripsacum Hybrids 

The 56-chromosomal apomictic hybrids isolated in BC2 were pollinated with tetraploid maize to test the stability of the expression of asexual reproduction in generations. The tetraploid pollinator allowed us to detect a deviation in chromosome number in progeny and to reject plants without apomixis. In addition, the tetraploid pollinator gives a higher yield of backcross progeny due to the reduced imprinting effect when using diploid pollen. As a result of these selections, it has now been established that only one of the 56-chromosomal hybrids with karyotype 2n = 56 = [(10Zm(573MB) + 36Td) + 10Zm(611CB)] stably expresses the trait of apomictic reproduction in a series of six generations. Another plant with karyotype 2n = 56 [(10Zm(573MB) + 36Td) + 10Zm(573MB)] formed unreduced ovules in 50% of the cases.

### 2.4. Selection of Apomictic 56-Chromosomal Plants with Fertile Pollen 

**Figure 4:**
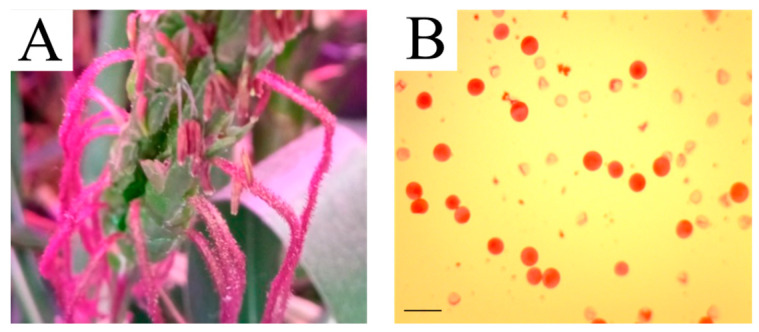
(**A**) Pollen on the stigmas of plant №VII-6; (**B**) the establishment of the pollen fertility of the №17CNR plant by the acetocarmine method. Scale bar = 200 µm.

Generations from the resulting apomictic 56-chromosome plant with the combination 2n = 56 = [(10Zm(573MB) + 36Td) + 10Zm(611CB)], as well as generations from a plant with karyotype 2n = 56 [(10Zm(573MB) + 36Td) + 10Zm(573MB)] (with unstable apomixis expression) were grown for several years. In 2020, fits of two plants (one plant from each combination) ejected anthers. By staining the pollen obtained from them according to the acetocarmine technique [24], we found that some of the pollen grains were fertile (Figure 4). The plants were named №17CNR (karyotype 2n = 56 = [(10Zm(573MB) + 36Td) + 10Zm(573MB)]) and №VII-6 (karyotype 2n = 56 = [(10Zm(573MB) + 36Td) + 10Zm(611CB)]). The self-pollination of №VII-6 x №VII-6 produced productive seeds, while no grains were formed in №17CNR x №17CNR. Plant №17CNR pollinated with the pollen of plant №VII-6 also set grains with accomplished endosperm. The plants grown from the resulting grains also formed fertile anthers.

### 2.5. Establishment and Analysis of Nucleotide Sequences of Pox3 and tRNA-Leu Genes in the Parental Lines and Hybrids

Molecular genetic methods were used to control the presence in the hybrid of the genomes of both maize lines used to create commercial seeds. For this purpose, the partial nucleotide sequences of the nuclear gene encoding peroxidase (*Pox3*) and the chloroplast *tRNA-Leu* (*trnL*) gene were determined in the parental lines *Z. mays*, *T. dactyloides*, and their hybrid (Table 4).

**Figure 5:**
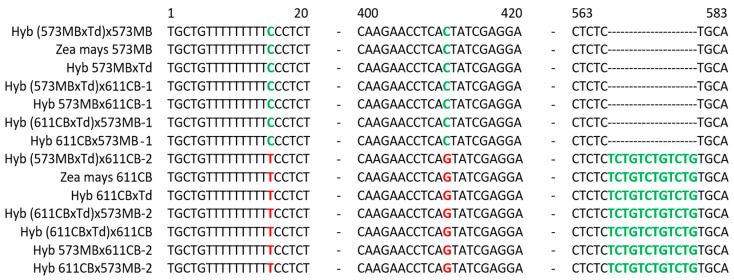
Partial sequence alignment of the *Pox3* gene in the original *Zea mays* lines 573MB and 611CB and their hybrids. Differences between the sequences are highlighted in color.

The main objective of the nucleotide sequence determination was to confirm the presence of the original nuclear genomes of the *Z. mays* lines 573MB and 611CB and *T. dactyloides* in the intergeneric hybrids. The sequences of the *Pox3* gene in lines 573MB and 611CB differ due to the presence of a 12-nucleotide deletion in the former (Figure 5). Based on the comparative analysis of the nucleotide sequences of the *Pox3* gene in the maize lines 573MB and 611CB and their hybrids with gamagrass, it was found that the nuclear genome of all the 56-chromosomal hybrids 2n = 56 = [(10Zm(573MB) + 36Td) + 10Zm(611CB)] contains variants of the *Pox3* gene from both the maize lines. This indicates that the genomes of the lines used in this experiment were successfully combined (Figure 5). The presence of the *Pox3* gene of *T. dactyloides* in the genomes of the hybrids was not investigated due to the absence of its nucleotide sequence in databases.

**Figure 6:**
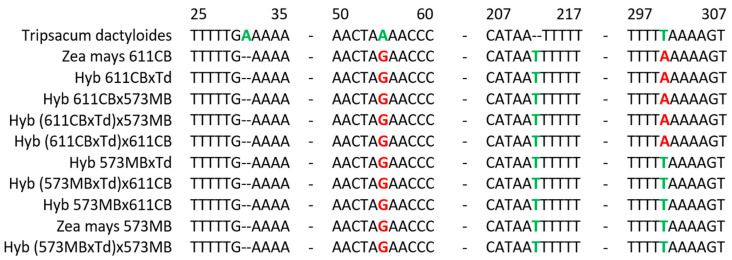
Partial sequence alignment of the *trnL* gene in the original lines *Zea mays* 573MB and 611CB, *Tripsacum dactyloides*, and their hybrids. Differences between the sequences are highlighted in color.

Figure 6 shows the alignment of a part of the chloroplast *trnL* gene from the original lines 573MB and 611CB, *T. dactyloides,* and their hybrids. The starting lines differ from each other by a single nucleotide substitution at position 400 (T in line 573MB and A in line 611CB). In turn, the read fragment of the *trnL* gene in *T. dactyloides* differed by three nucleotide substitutions from the *trnL* gene of both *Zea mays* lines. As a result of this work, it was found that the *trnL* gene corresponded to the maternal variant in all the hybrids studied.

## 3. Discussion

Distant crosses, with the purpose of transferring economically valuable traits from wild relatives to cultivated plants, have long been successfully used in breeding practice [25]. But, the analysis of such studies conducted on maize leads to a paradoxical conclusion. Despite the wide spread and economic importance of this crop, only two resistance genes from its closest relative—teosinte [26] and only one from gamagrass [27] have been introduced to date. At the same time, there is no evidence that the materials obtained have been further utilized in the development of commercial hybrids. At the same time, in other crops, dozens of varieties have been developed that have received resistance genes from wild relatives, both to infectious diseases and to unfavorable environmental impacts [26]. This imbalance between the economic importance of maize and the lack of achievements from the use of wild relatives in its practical breeding is due to several circumstances.

Historically, the vast majority of first-cycle breeding lines have been derived from the use of single varieties from the U.S. Corn Belt. This raised concerns about the possible genetic erosion of the crop. Therefore, in the mid-20th century, numerous expeditions to Mexico and other Latin American countries were undertaken by U.S. geneticists and breeders to collect, describe, multiply, catalog, and preserve the germplasm of a large number of maize accessions. Unfortunately, only 3–8% of this diversity [28] has been involved in the breeding process.

Practically until the end of the 20th century, the main world producer of marketable maize grain and seeds was the USA, where the technologies of disease and pest control with the help of the chemical means of protection have been developed and there is a high-tech chemical industry. Therefore, with great attention to maize as a genetic object, the works on distant hybridization were very fragmentary and academic in nature. In addition, these studies are very labor-intensive and require considerable efforts from specialists of different specialties, as well as field work with large volumes of plant material.

Currently, more than half of the world’s maize crop is produced in China and Latin America, and maize cultivation is expanding in Africa, which has created a need to develop varieties that are resistant to biotic and abiotic environmental factors that are new to the crop. A good way out of this situation could be the use of interspecific hybridization, which allows us to go beyond the species, along with the use of the potential of the intraspecific variability of maize [29]. Gamagrass is particularly interesting from the point of view of alien plasmid recruitment because it and maize do not share infectious diseases and hence could be a source of resistance genes. In addition, *Tripsacum* can transmit tolerance to unfavorable environmental factors and parasitic plants [30,31,32]. Apparently, in the near future, the work on obtaining distant hybrids of maize will receive a new impetus, since several laboratories in China have started and actively, at a good level, conduct research on this problem [29,33,34,35].

Despite the attractiveness of *Tripsacum* as a source of genes for host-valuable traits and, in particular, apomictic reproduction, as mentioned above, almost none of its genes have been introduced into maize. Based on the results of a previous study [22], we understood that apomixis in *Zea mays* suffers from epigenetic stresses and it would not be possible to transfer it to maize through chromosome recombination. However, seeing the result of the successful fixation of apomixis in 56-chromosome maize–*Tripsacum* hybrids [14], we assumed that the fixation of apomixis is possible in hybrids, and therefore, we used the same parental forms for crossing. When planning the volumes of crosses for obtaining hybrids when pollinating maize with the pollen of *Tripsacum*, we proceeded from the results described earlier [36]. But at the same time, knowing the difficulty in controlling this trait, we conducted an order of magnitude larger number of pollinations. Here, we proceeded from the fact that our task was to transmit a trait controlled by at least six major genes and several minor genes [6]. If we ignore the latter, the probability of the main apomixis genes entering one sperm in the absence of linkage and transmitting them to the hybrid is 1 case out of 4096 or 0.02%. In this case, the complex of major genes includes those that control diplosporia, the parthenogenetic development of the unfertilized egg, and endosperm formation with imprinting blocking.

A total of 166,261 flowers of maize lines were pollinated with gamagrass pollen. Due to such a significant number of pollinated flowers, we were able to obtain sufficiently extensive source material for selection. To determine the apomictic nature of the obtained plants, we counted the number of chromosomes in the progeny of the obtained forms. This method, although more time-consuming relative to the analysis of callose deposition during megasporogenesis [20], is still, in our opinion, more reliable, as it allowed us to quickly identify forms with karyotypes different from the 46-chromosome or 56-chromosome plants, which indicated natural hybridization with tetraploid maize. In addition, our sequencing of kernel and chloroplast genes confirmed the presence of only the genomes of the lines 611CB and 573MB in the corresponding hybrids, which excluded the introduction of genetic material from tetraploid maize and confirmed the apomictic mode of reproduction. In aggregate, all of the above allowed us to identify several apomictic plants with both 46 chromosomes and 56 chromosomes.

The scheme for obtaining apomictic hybrids in our work is very similar to the first steps of the scheme for obtaining 38-chromosome maize–*Tripsacum* hybrids described in [22]. However, unlike the published experiment, where researchers developed an apomictic polyhaploid (2n = 38) combining haploid sets of chromosomes from two maize and *Tripsacum* lines, we continued to work with the resulting forms to restore the fertility of their anthers. As previously shown, a significant influence on the apomixis formation of maize–*Tripsacum* hybrids is exerted by epigenetic marks that alter the state of chromatin in the different tissues of the flower [37]. We hypothesized that pollen sterility, even in plants with a 56-chromosome karyotype (where normal conjugation should take place), may be determined by the similar mechanisms of chromatin state inactivation. Epigenetic marks are known to be reversible [38], so we hypothesized that in the subsequent generations of apomictic plants, forms with altered epigenetic marks may appear, which may affect anther productivity.

A few plant generations later, in 2020, fits of two plants moved from the field to the greenhouse ejected anthers with partially fertile pollen. At present, it is not known what causes the fertility of the anthers of these two plants. It is likely that the change in environmental conditions when the plants were transferred to the greenhouse may have affected the epigenetic marks in the regions of the genome responsible for the development of male inflorescences. The progeny of these two plants reproduced, and a significant proportion of them produced functional pollen. Nevertheless, it should be noted that the hybrids obtained are not superior in green mass productivity to the control 56-chromosome hybrids obtained by repeated pollination with the same inbred form (unpublished results). Apparently, the interactions between gamagrass and maize genomes have a stronger effect on plant habitus than the interactions between the genomes of maize inbred lines. However, our study and the plant material obtained in it may serve as a basis for obtaining new commercial varieties of grain-forage direction. Currently, Guatemala grass (*Tripsacum andersonii*) [39], which is a natural 64-chromosomal hybrid of maize or teosinte with gamagrass [11] (2n = 10Zm + 54Td), is widely used as a forage plant. As a forage plant, it has a number of useful traits that determine its sustainability and spread, while providing a source of high-quality forage for ruminants. Therefore, despite its complete male sterility, it is propagated vegetatively and is widely distributed in regions with inexpensive labor. The introduction of our hybrid-producing fertile seeds would make it much easier to produce high-quality forage.

## 4. Materials and Methods

### 4.1. Source Material and Hybridization

As parental forms for hybridization were taken the following: 1. *Zea mays* (Zm) (2n = 20)—lines 573MB and 611CB, the selection of NPO “KOS-Mays”, used in the production of commercial hybrid Kubansky 601; 2. Eastern gamagrass—*Tripsacum dactyloides* (Td) (2n = 4x = 72)—from the collection collected by N. I. Vavilov in Mexico. Hybridization was carried out with the preliminary pruning of corn stigmas [36]. To avoid self-pollination, male inflorescences in maize were removed as soon as they appeared, and the cobs were covered with insulators made of moisture-resistant paper to prevent accidental pollination. The establishment of the 56˗chromosomal hybrids was carried out in two stages. In the first step, parental maize lines were pollinated with pollen from gamagrass ♀*Z. mays* × ♂*T. dactyloides*. The resulting F1 (573MB × Td) and F1 (611CB × Td) intergeneric hybrids were 46˗chromosomal forms with the genomic combination 2n = 46 = (10Zm + 36Td). Next, the apomictic 46˗chromosomal plants were selected from these and backcrossed with the parental maize lines BC1 (♀F1 × ♂573MB) and BC1 (♀F1 × ♂611CB). In this case, BIII hybridization resulted in the 56˗chromosomal forms 2n = 56 = [(10Zm + 36Td) + 10Zm], where genomes from the 573MB and 611CB lines were combined.

The production of 46- and 56-chromosomal maize–*Tripsacum* hybrids, their apomictic testing, and the propagation of apomictic plants were conducted at the Kuban Experimental Station of VIR (45°21′ N latitude and 40°79′ E longitude, Botanika village, Krasnodar Krai, Russia) from 2012 to 2017. Every year in the third decade of March, the seeds of 46- and 56-chromosome plants were placed to germinate in a thermostat at +26 °C. The germinated seeds were planted in cups filled with prepared potting soil and grown in the growing house until the end of April, after which they were transferred to the field. The experiments were conducted using a randomized block design in four replications. Plots were divided into 5 m double rows with 48 individuals living in them [40]. The seedlings of the 46-chromosome hybrids were planted at the 4–5 leaf stage with a distance of 1 m between plants and 1 m between rows; the seedlings of the 56-chromosome hybrids were planted at the 5–7 leaf stage with a distance of 1.4 m between plants and 1.4 m between rows. The maize lines 573MB and 611CB were sown together with hybrids to pollinate the 46-chromosome forms, and the tetraploid maize was sown to pollinate the 56-chromosome plants. Pollination with diploid pollen gives a greater seed set compared to haploid pollen. Pollinators were planted at five or six dates with an interval of seven days.

### 4.2. The Method of Determining Pollen Fertility

The fertility of the pollen grains was determined using a standard acetocarmine technique [24]. The pollen grains were placed on a slide on the surface of a drop of acetocarmine and covered with a cover glass. The preparation was heated over an alcohol lamp for 3 to 5 s. The pollen grains were examined using an Olympus BX53 microscope at magnification 200×. The fertility of the pollen grains was determined by their color: if pollen grains were dark red and burgundy in color they were classified as fertile. The number of fertile and sterile grains in 10 fields of view of the microscope was calculated on each preparation.

### 4.3. Determination of Modes of Reproduction

To establish the presence of apomictic reproduction, the chromosome counting of the analyzed plants was carried out. Since the obtained plants had a karyotype with 46 chromosomes or 56 chromosomes, pollination with the tetraploid maize allowed us to quickly detect a deviation in the number of chromosomes and to reject the forms without apomixis. Preparations were prepared as follows: the root tips of the maize–*Tripsacum* hybrids were pretreated with α-bromonaphthalene at room temperature for 3 h, then fixed in glacial acetic acid for 15–20 min. This was followed by hydrolysis in 1N hydrochloric acid for 1 h at +60 °C. The pressed preparations were prepared in a drop of aceto-orcein on a slide; the chromosome numbers were counted at the metaphase stage at a magnification of 100 × 20 on an Olimpus BX53 microscope.

### 4.4. Molecular–Biological Testing of Hybridization Results: Total DNA Isolation, PCR Amplification and Nucleotide Sequence Determination

Total DNA was isolated using the DNeasy Plant Mini Kit (QIAGEN, Hilden, Germany) according to the manufacturer’s protocol. For DNA extraction, 50–100 mg of freshly harvested leaves obtained from the plants of each sample were used. The quantity and quality of the isolated DNA was determined using a NanoDrop2000 spectrophotometer (Thermo Scientific, Waltham, MA, USA) and electrophoretic separation in 1% agarose gel containing ethidium bromide (0.5 mg/mL) in 1xTAE. PCR amplification was performed as described in [41]. The PCR products were cloned into the pAL2-T vector according to the protocol of the Quick-TA kit (Eurogen, Moscow, Russia).

Sequencing reactions were performed using 200 ng of product and the BigDye Terminator v3.1 Cycle Sequencing Kit (Thermo Scientific, USA) on an ABI 3130XL genetic analyzer (Applied Biosystems, Waltham, MA, USA) at the Genomics Center of SB RAS (http://www.niboch.nsc.ru/doku.php/corefacility accessed on 20 June 2023).

## 5. Conclusions

In this work, we obtained 56-chromosome maize–*Tripsacum* hybrids with the apomictic type of reproduction. Apomixis was confirmed by cytological methods and the sequencing of nucleus and chloroplast genes. It was also possible to obtain two populations of apomictic 56-chromosomal plants with fertile anthers derived from two plants №17CNR and №VII-6. The hybrids obtained are of great scientific value and can be the basis for obtaining commercial varieties of forage plants with fixed heterosis.

## Figures and Tables

**Table 1:** Hybridization results of lines 573MB and 611CB with *Tripsacum dactyloides*.

Maize Lines, Pollinated *T. dactyloides*	Number of Pollinated Flowers, pcs *.	Number of Developed Embryos, pcs.	Number of F1 Grains That Formed Endosperm, pcs.	Number of F1 Hybrid Plants Grown, pcs.
573MB	103,163	28,193	909	429
611CB	68,998	29,840	31	3

* pcs—pieces.

**Table 2:** Percentage of formed grains at pollination of 46 chromosomal hybrids by maize initial lines.

Scheme of Backcross 1	Number of Pollinated Plants, pcs *	Number of Developed Embryos, pcs.	Number of BC1 Grains That Formed Endosperm, pcs.	Percentage of Formed Grains, %
♀(573MB × Td) × ♂573MB	11	2255	23	1.02
	9	1467	277	18.88
♀(573MB × Td) × ♂611CB	11	5359	40	0.75
	9	3412	663	19.43
♀(611CB × Td) × ♂573MB	2	409	6	1.47
♀(611CB × Td) × ♂611CB	2	600	18	3

* pcs—pieces.

**Table 3:** Results of the backcrossing of the F1 hybrids with the 573MB and 611CB lines.

№	Hybrid Combination F1 *Z. mays × T. dactyloides*	№ Original F1	Pollinator Line	Number of Grains Received BC1	Number of Plants Obtained BC1 ♀F1 × ♂Zm			Behavior of 56-ch. Hybrids in BC2
					46-ch., pcs.	56-ch., pcs. (% of All Plants)	Of These BC1_apo_ 56-ch., pcs.	
1	(573MB *×* Td)	1/Б10-9	573MB 611CB	24 91	14 61	1 (6.7%) 1 (1.6%)	0 0	No progeny No progeny
2	(573MB *×* Td)	3/Б10-1	573MB 611CB	11 12	9 3	1 (10%) 3 (50%)	0 0	No progeny B_III_-hybridization
3	(573MB *×* Td)	3/Б10-8	573MB 611CB	55 233	12 136	1 (7.7%) 4 (2.9%)	1 4	Stable 56-ch. Stable 56-ch.
4	(573MB *×* Td)	3/Б10-9	573MB 611CB	16 11	0 0	6 (100%) 8 (100%)	0 0	B_III_-hybridization B_III_-hybridization
5	(573MB *×* Td)	4/Б10-9	573MB -	49 -	37 -	0 -	0 -	- -
6	(611CB *×* Td)	26/Б11-1	573MB 611CB	3 11	2 6	0 1 (14.3%)	0 1	- Stable 56-ch.

**Table 4:** Results of determining the variants of nuclear *Pox3* and chloroplast *trnL* genes in the initial lines (573MB and 611CB) of *Z. mays*, *T. dactyloides*, and their hybrids. In the hybrids, the maternal parent is listed first in the hybrid name. Td—*T. dactyloides*.

№	Accession or Hybrid	Sample Karyotypes, 2n	*Pox3*	*trnL*
1	*T. dactyloides*	72	-	Td
2	*Zea mays* line 573MB	20	573MB	573MB
3	Hybrid F1 573MB × 611CB	20	573MB + 611CB	573MB
4	Hybrid F1 573MB × Td	46	573MB	573MB
5	Hybrid BC1 (573MB × Td) × 573MB	56	573MB	573MB
6	Hybrid BC1 (573MB × Td) × 611CB	56	573MB + 611CB	573MB
7	*Zea mays* line 611CB	20	611CB	611CB
8	Hybrid F1 611CB × 573MB	20	611CB + 573MB	611CB
9	Hybrid F1 611CB × Td	46	611CB	611CB
10	Hybrid BC1 (611CB × Td) × 611CB	56	611CB	611CB
11	Hybrid BC1 (611CB × Td) × 573MB	56	611CB + 573MB	611CB

## Data Availability

The nucleotide sequences of the *pox3* and *trnL-UAA* genes are provided in GenBank (OR834333-OR834346 *pox3* gene, OR791522-OR791532 *trnL-UAA* gene).
